# Aberrant promoter methylation of *PPP1R3C* and *EFHD1* in plasma of colorectal cancer patients

**DOI:** 10.1002/cam4.273

**Published:** 2014-05-24

**Authors:** Kiyoko Takane, Yutaka Midorikawa, Koichi Yagi, Ayako Sakai, Hiroyuki Aburatani, Tadatoshi Takayama, Atsushi Kaneda

**Affiliations:** 1 Department of Digestive Surgery and Pathology, Nihon University School of MedicineTokyo, Japan; 2 Department of Molecular Oncology, Graduate school of Medicine, Chiba UniversityChiba, Japan; 3 Genome Science Division, Research Center for Advanced Science and Technology, The University of TokyoTokyo, Japan; 4 Department of Gastrointestinal Surgery, Graduate School of Medicine, The University of TokyoTokyo, Japan; 5 Sysmex CorporationKobe, Japan; 6 CREST, Japan Science and Technology AgencySaitama, Japan

**Keywords:** Biomarker, cancer detection, colorectal cancer, DNA methylation, plasma DNA

## Abstract

Aberrant DNA methylation is a common epigenetic alteration involved in colorectal cancer (CRC). In our previous study, we performed methylated DNA immunoprecipitation-on-chip analysis combined with gene re-expression analysis by 5-aza-2′-deoxycytidine treatment, to identify methylation genes in CRC genome widely. Among these genes, 12 genes showed aberrant hypermethylation frequently in >75% of 149 CRC samples but did not in normal samples. In this study, we aim to find out any of these methylation genes to be utilized for CRC detection using plasma DNA samples. Primers for methylation-specific PCR and pyrosequencing were designed for seven of the 12 genes. Among them, *PPP1R3C* and *EFHD1* were rarely hypermethylated in peripheral blood cells, but frequently hypermethylated in 24 CRC tissue samples and their corresponding plasma samples. In plasma samples, *PPP1R3C* was methylated in 81% (97/120) of CRC patients, but only in 19% (18/96) of noncancer patients (*P* = 6 × 10^−20^, Fisher's exact test). In combined analysis with *EFHD1*, both genes were methylated in 53% (64/120) of CRC patients, but only in 4% (4/96) of noncancer patients (*P* = 2 × 10^−16^), giving high specificity of 96%. At least one of the two genes was methylated in 90% (108/120) of CRC patients, and 36% (35/96) of control patients, giving high sensitivity of 90%. Compared with low sensitivity of carcinoembryonic antigen (17% at stage I, 40% at stage II) and CA19-9 (0% at stage I, 13% at stage II) for early-stage CRCs, sensitivity of aberrant methylation was significantly higher: *PPP1R3C* methylation at 92% (11/12) for stage I and 77% (23/30) for stage II, and methylation of at least one gene at 100% (12/12) for stage I and 87% (26/30) for stage II. *PPP1R3C* methylation or its combined use of *EFHD1* methylation was highly positive in CRC plasma samples, and they might be useful in detection of CRC, especially for early-stage CRCs.

## Introduction

For cancer treatment, early detection of disease leads to favorable outcomes for patients, and it is important to develop screening tests with high sensitivity and specificity, especially for early-stage cancer 1. In colorectal cancer (CRC) screening, stool blood tests and measurement of tumor markers in serum, such as carcinoembryonic antigen (CEA) and carbohydrate antigen (CA19-9), are conventional methods that have been used. The fecal occult blood test, however, has a low specificity, ranging from 0.3% to 0.5% 2. CEA and CA19-9 are not frequently positive in CRC at early stages (I and II), and their sensitivities are <50% 3. These methods are not satisfactory for early cancer detection, and a new, noninvasive technique to detect early-stage malignancies with higher sensitivity than these protein markers would be useful as a first screening test, before the need of invasive examinations, for example, barium enemas and colonoscopies 4,5.

Cell-free DNA derived from solid tumor cells circulates in the blood stream; therefore, detection of tumor DNA in plasma/serum could be an attractive method for cancer screening 6. For example, detection of mutated *RAS* gene fragments 7 and microsatellite aberrations 8 in plasma/serum of cancer patients have been demonstrated. But these methods can detect only a fraction of cancer cases with specific genomic aberrations such as *RAS* mutations, and the development of screening methods to detect the majority of cancer cases are urgently needed. Aberrant DNA methylation of promoter CpG islands is a common epigenetic alteration to inactivate tumor suppressor genes in CRC and in other cancers 9,10. Detection of genetic mutations is rather difficult to apply to cancer screening because it is necessary to examine many possible mutation sites per gene. When DNA methylation is analyzed, only one promoter region per gene needs to be examined.

In detection of aberrantly methylated DNA in plasma samples, Lofton-Day et al. identified three blood-based molecular biomarkers including *TMEFF2*, *NGFR*, and *SEPT9* that were useful for CRC screening 11. Thereafter, the concentration of *SEPT9* methylated DNA could be measured with higher sensitivity and specificity and detected in a majority of CRCs at all stages and colorectal locations 12.

A subgroup of CRC shows aberrant CpG island methylation at a significantly higher frequency, which is called CpG island methylator phenotype (CIMP) 13,14. We 15 and other groups 16–18 performed comprehensive methylation analysis of CRC samples and reported three distinct DNA methylation epigenotypes of CRC: high-, intermediate-, and low-methylation epigenotypes. In the analysis, we performed methylated DNA immunoprecipitation-on-chip analysis of CRC cell lines combined with microarray analysis of gene re-expressions by 5-aza-2′-deoxycytidine treatment, and established methylation genes to epigenotype CRC 15. These epigenotyping genes included two major groups of genes: Group-1 genes specifically methylated in high-methylation/CIMP(+) CRCs and Group-2 genes methylated in both high- and intermediate-methylation CRCs. These genes therefore classify CRC into three epigenotypes: high-methylation/CIMP(+) CRCs with methylation of Group-1 and Group-2 genes, intermediate-methylation CRCs with methylation of Group-2 genes, and low-methylation CRCs without methylation of either group of genes. Besides these genes, another type of genes was found to be hypermethylated in all or most CRC cases regardless of epigenotype 15.

In this study, we aim to find out whether any of these commonly hypermethylated genes could be utilized for CRC detection using plasma DNA samples. For candidate genes showing aberrant methylation in >75% of CRC samples but in none of normal samples in the previous analysis, we first checked methylation status of peripheral blood cells. Genes rarely methylated in peripheral blood cells underwent subsequent methylation analysis using plasma DNA samples of CRC and noncancer patients. Methylation was analyzed using methylation-specific PCR 19 in conjunction with pyrosequencing 20, which was used for the validation of the methylation-specific amplification. It was found that *PPP1R3C* methylation alone or in combination with *EFHD1* methylation showed high sensitivity and specificity, and these genes could be used to detect CRC, especially at early stage.

## Material and Methods

### Clinical samples

Peripheral blood was collected from 96 patients undergoing surgical operations for benign diseases including inguinal hernia, appendicitis, and gallbladder stones (noncancer group), and from 120 patients undergoing surgical operations for CRC (CRC group). Corresponding primary CRC tissue samples were also collected from 24 CRC patients. All samples were collected with written informed consent and the surgery was done in the Department of Digestive Surgery, Graduate School of Medicine, Nihon University. Tissue samples were immediately frozen in liquid nitrogen and stored at −80°C. Frozen materials were microscopically examined for the determination of cancer cell content by pathologists, and it was confirmed that all 24 samples contained at least 40% cancer cells. DNA was extracted using QIAamp DNA Mini Kit (Qiagen, Valencia, CA) according to the manufacture's protocol. Peripheral blood was put in an ethylenediaminetetraacetic acid vacutainer coated tube and centrifuged at 1200*g* at room temperature for 15 min. From 3 mL of the supernatant plasma, cell-free genomic DNA was extracted using QIAamp Circulating Nucleic Acid Kit (Qiagen). The Ethics Committees of Nihon University, Chiba University, and The University of Tokyo certified this study.

### Characteristics of the study population

The 120 CRC patients were 67.7 ± 11.4 years old (mean ± standard error), ranging 30–88, and included 71 males and 49 females, whereas the 96 noncancer patients were 63.0 ± 13.6 years old, ranging 24–87 (*P* = 1, *t*-test vs. CRC patients), and included 67 males and 29 females (*P* = 0.1, Fisher's exact test vs. CRC patients). Twenty (17%) CRC patients underwent neoadjuvant chemotherapy. As for tumor location, 41 (34%) were at proximal colon (10 in cecum, 15 in ascending colon, 16 in transverse colon), 37 (31%) at distal colon (4 in descending colon, 33 in sigmoid colon), and 42 (35%) at rectum. For AJCC (American Joint Committee on Cancer) stages, 12 (10%) were at stage I, 30 (25%) at stage II, 12 (10%) at stage III, and 66 (55%) at stage IV.

### Bisulfite treatment of genomic DNA

By bisulfite treatment, unmethylated cytosine is converted to uracil—that is, recognized as thymine (T) after PCR, but methylated cytosine is not converted—that is, recognized as cytosine (C) after PCR. Unmethylated DNA and methylated DNA are therefore distinguishable by detecting the difference of T and C in the sequence after bisulfite treatment. Bisulfite conversion of 500 ng of genomic DNA from each tissue sample was performed using Zymo EZ DNA Methylation Kit (Zymo Research, Irvine, CA), and the DNA was eluted in 30 *μ*L of 10 mEq Tris buffer. For plasma samples, genomic DNA isolated from 3 mL of plasma was treated with bisulfite in the same manner. To check the quality of bisulfite-converted DNA sample as PCR template, 5.3 kb upstream region of *MYOD* (chr11:17,735,751-17,735,847) 21 was amplified by PCR and the PCR product was visualized using ethidium bromide after agarose gel electrophoresis. Primers for *MYOD* were 5′-TGATTAATTT AGATTGGGTT TAGAGAAGGA-3′ (forward) and 5′-CTCCCTCTAT CCCCTAACAA ACTT-3′ (reverse). PCR product length was 97 bp and annealing temperature was 62°C. This region contains no CpG site, and should therefore be amplified regardless of methylation status.

Methylation control samples (0% and 100%) were prepared as previously described 15. Briefly, human peripheral lymphocyte DNA was amplified using GenomiPhi v2 DNA amplification kit (GE Healthcare Life-Science, Uppsala, Sweden). The amplified DNA was not methylated at all in any CpG sites, and was used as unmethylated (0%) control. The amplified DNA was methylated by *Sss*I methylase and used as fully methylated (100%) control. These control samples were also treated with bisulfite using Zymo EZ DNA Methylation Kit.

### Methylation-specific PCR

Methylation status was determined by methylation-specific PCR 19. To design primers, Pyro Q-CpG software (Qiagen) was used to obtain the genomic DNA sequence after bisulfite conversion, by converting C at non-CpG sites to T and retaining C at CpG sites as C. Forward and reverse primers were designed to contain multiple C's, especially at the 3′ end of primer. When annealing temperature is high enough, the primers would anneal to methylated allele only, and unmethylated allele containing T at CpG sites should not be recognized and amplified.

Methylation genes in CRC were selected from genes identified in our previous study 15, in which bisulfite sequencing primers were designed in the 5′ region of each gene. The PCR products were 200–400 bp, and were analyzed in the methylation assay using MALDI-TOF-MS (matrix-assisted laser desorption ionization–time-of-flight–mass spectrometry) 22. In this study, primers for methylation-specific PCR were designed within these regions, with PCR products being ≤100 bp, because these analyzed regions were located in 5′ CpG islands of genes and confirmed to be aberrantly methylated in CRC. PCR was performed using 5 *μ*L of bisulfite-modified DNA as a template, and FastTaq polymerase (Roche, Basel, Switzerland). The annealing temperature for the PCR was determined to amplify 100% methylation control sample only, and not to amplify 0% methylation control sample. Among 12 candidate genes, *COL4A2*, *TSPYL5*, *TMEFF2*, *RASSF2*, *SPG20*, *EDIL3*, *CIDEB*, *ADAMTS1*, *EFHD1*, *STOX2*, *PPP1R3C*, and *UCHL1*, such primers could be designed for seven genes, *COL4A2*, *TSPYL5*, *EDIL3*, *ADAMTS1*, *EFHD1*, *STOX2*, and *PPP1R3C*. Primer sequences for these genes and the number of analyzed CpG sites are shown in Table 1.

**Table 1:** Primer sequences for methylation-specific PCR and for pyrosequencing

Primer sequence	Anneal (°C)	Product (bp)	Number of analyzed CpG sites	Primer position
*ADAMTS1* (*Bottom strand*)				
Fwd: GTTTCGAGATTTCGGAGTTCGTTTCGC	64	97	5	+538 to +512
Rev^1^: AAACTCCAATACAACGAACTATACCCG			2	+470 to +442
Seq: TTTTTTATGTAGTTGTTTAGTT			2	+510 to +499
*STOX2* (*Top strand*)				
Fwd^1^: TGGGGTAGTTGTTAAGGTTTTCGCGTC	61	97	3	+301 to +327
Rev: CACCAAACTACCTTAAATTAAAACGCG			2	+371 to +397
Seq: CATCAAACTTCTCATTTTCATATA			4	+375 to +352
*EDIL3* (*Bottom strand*)				
Fwd: GATTAAGAGTTAGACGGTTATCGAGC	64	79	3	+452 to +427
Rev^1^: CGCGACGACCCCTAACCAACCGAAATCACG			5	+403 to +374
Seq: GGTTATAGAGAGTTTTATGATTT			2	+437 to +415
*COL4A2* (*Bottom strand*)				
Fwd: TTTATCCTCGGTTTCGGTTC	64	72	3	+529 to +510
Rev^1^: CTCCCATCACCCCTACATACG			1	+478 to +458
Seq: GAGAAGAGGGGATAG			4	+507 to +493
*PPP1R3C* (*Top strand*)				
Fwd: TCGTTTCGGGGCGATTACGTTGTC	65	100	5	−123 to −120
Rev^1^: CCTAAAACCAATCGCCGAACCTCG			3	−47 to −24
Seq: GAGGGTTGGAGTTTTAGTTGG			3	−114 to −94
*EFHD1* (*Top strand*)				
Fwd: TTTCGAGTTTGCGAGGAGCGCGTC	68	90	5	+4 to +27
Rev^1^: CATAACGACGAATCGCAAAACGCG			5	+70 to +93
Seq: CGTCGTTAGTTAGTTTTTTG			6	+24 to −43
*TSPY5* (*Top strand*)				
Fwd: TATAGTTGTACGTTCGTGACGTC	61	75	4	−17 to +6
Rev^1^: CCTAACGCCAACTCTCGATCG			3	+38 to +58
Seq: GGTTGTAGTGGAGAGATT			4	+10 to +27

The position of the transcription start site (TSS) was regarded as +1. The DNA strand used for the template was shown by *top/bottom*. *Fwd/Rev*, forward and reverse primers for methylation-specific PCR; *Seq*, sequence primer for pyrosequencing; *C/G*, C in forward primer and G in reverse primer to distinguish methylated DNA from unmethylated DNA.

1 Primers biotinylated for pyrosequencing.

### Pyrosequencing analysis

To confirm that methylation-specific PCR specifically amplified the methylated allele, the methylation status of the PCR product was quantitatively sequenced using pyrosequencing as previously described 23. Briefly, the biotinylated PCR product was bound to streptavidin Sepharose beads HP (GE Healthcare Life Sciences), washed and denatured using a 0.2 mol/L NaOH solution. After addition of 0.3 *μ*mol/L sequencing primer to the purified, single-stranded PCR product, pyrosequencing was carried on PyroMark Q24 MD System (Qiagen) with Pyro Q-CpG software (Qiagen) according to the manufacturer's instructions. Primer sequences and conditions, and the number of analyzed CpG sites are shown in Table 1. Methylation control samples (0% and 100%) were analyzed in every assay to check that no PCR product was obtained in the 0% control sample and that the fully methylated allele was amplified in the 100% control sample.

### Evaluation of protein markers CEA and CA19-9

At clinical diagnosis of CRC, serum CEA and CA19-9 levels were evaluated by Enzyme-linked immunosorbent assay. CEA and CA19-9 were considered positive when CEA was ≥5 ng/mL and CA19-9 was ≥40 U/mL.

### Statistical analysis

*P*-values were calculated to compare CRC patients and noncancer patients. Student's *t*-test was used for age and Fisher's exact test was used for analysis of sex. *P*-values were also calculated to compare methylation(+) group and methylation(−) group. Student's *t*-test was used for age and Fisher's exact test was used for analysis of sex, AJCC stage, neoadjuvant chemotherapy, and tumor locations (Tables 2 and 3). In each AJCC stage, methylation frequency in plasma DNA samples was also compared with frequencies of CEA(+) and CA19-9(+) using Fisher's exact test (Fig.6). When *P* < 0.05, the correlation was considered statistically significant. Student's *t*-test and Fisher's exact test were performed using R software (http://www.r-project.org/).

**Table 2:** *PPP1R3C* methylation and clinicopathological factors

	Methylated	Unmethylated	*P-*value
Number	97	23	
Age (years)	67.9 ± 11.4	67.0 ± 11.8	0.9
Sex (male/female)	59/38	12/11	0.7
AJCC stage			0.7
I/II/III/IV	11/23/9/54	1/7/3/12	
NAC (yes/no)	17/80	3/20	0.5
Tumor location			0.4
Proximal (Ce/A/T)	36 (9/11/16)	5 (1/4/0)	
Distal (D/S)	29 (3/26)	8 (1/7)	
Rectum	32	10	

Age was shown by mean ± standard deviation. AJCC, American Joint Committee on Cancer; NAC, neoadjuvant chemotherapy. Tumor locations were classified into proximal colon including cecum (Ce), ascending (A) and transverse colon (T), distal colon including descending (D) and sigmoid colon (S), and rectum. *P-*values were analyzed using the Student's *t*-test for age and the Fisher's exact test for sex, stage, NAC, and tumor location.

## Results

### Selection of candidate genes

**Figure 1:**
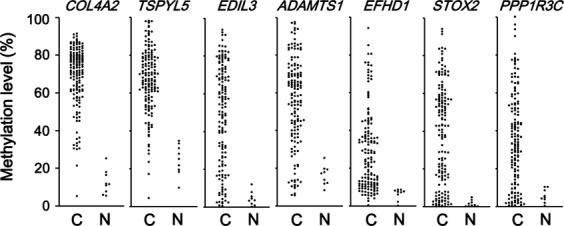
Candidate genes frequently hypermethylated in CRC. Using quantitative methylation data from 149 CRC and nine normal colon samples conducted in our previous analysis 15, we selected 12 genes that were not hypermethylated in normal colon (methylation level <35% in all nine samples), but aberrantly methylated in most of the 149 CRC cases (methylation level >35% in 112 or more samples). Seven genes, for which primers could be designed, are representatively shown.

In our previous methylome analysis of CRC, 60 methylation genes to epigenotype CRC were established and their methylation levels were analyzed quantitatively in 149 CRC and nine normal colon samples 15. Among them, 12 genes were not hypermethylated in any of the normal colon samples, but were frequently methylated (>75%) in CRC cases: *COL4A2* (147/149), *TSPYL5* (141/149), *TMEFF2* (141/149), *RASSF2* (134/149), *SPG20* (130/149), *EDIL3* (130/149), *CIDEB* (128/149), *ADAMTS1* (128/149), *EFHD1* (127/149), *STOX2* (126/149), *PPP1R3C* (118/149), and *UCHL1* (115/149) (Fig.1). *CDO1*, *SFRP1*, and *PENK1* showed frequent hypermethylation in >75% of CRC cases, but were also aberrantly methylated in normal samples. Although the size of the normal samples was as small as nine, the former 12 genes were extracted as candidate genes because of no hypermethylation in normal samples, and the latter three genes were excluded.

To detect aberrantly methylated alleles, bisulfite-treated genomic DNA was amplified using methylation-specific PCR primers designed to generate PCR products ≤100 bp. To validate that methylation-specific PCR products resulted from amplification of methylated alleles, and not from unexpected amplification of unmethylated DNA or DNA with partial methylation in primer regions, sequence primers were designed within the product regions and the methylation level of the PCR products were analyzed using pyrosequencing. Such primers for methylation-specific PCR and pyrosequencing were successfully designed for seven of the 12 genes: *COL4A2*, *TSPYL5*, *EDIL3*, *ADAMTS1*, *EFHD1*, *STOX2*, and *PPP1R3C* (Table 1).

In pyrosequencing, the signal intensity should be high enough (≥5) and detected methylation rate should be high enough (60–100%) if methylated allele was successfully amplified. If methylation rate were low (<60%), that would be due to unexpected amplification of unmethylated allele in methylation-specific PCR, and the sample would therefore be regarded as methylation(−). But all the analyzed samples showed methylation rate as high as 60–100% when the signal intensity was higher than 5, and they were regarded as methylation(+). When no signal was detected in pyrosequence, that should be due to no amplification in methylation-specific PCR, the sample was regarded as methylation(−). When the signal intensity was too low to accurately calculate methylation rate, that would be regarded as insufficient amplification by methylation specific PCR, we set the threshold of the signal intensity at 5; the sample was regarded as methylation(−) when the signal intensity was <5 (Fig. S1). To check the quality of sample DNA, bisulfite-converted DNA was amplified using primers for *MYOD* upstream region. *MYOD* primers were designed in the regions without CpG sites, and therefore amplify the region regardless methylation status. All the analyzed samples showed amplification of the *MYOD* region, indicating that lack of amplification is due to absence of methylation, not due to poor DNA quality (Fig. S1).

### Selection of genes using normal peripheral blood cell samples

**Figure 2:**
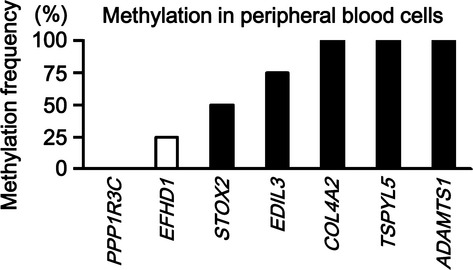
Screening of seven genes using peripheral blood cells. Methylation status was analyzed using peripheral blood cell samples from four noncancer patients. *PPP1R3C* and *EFHD1* showed no or infrequent methylation (*open box*), but the other five genes showed methylation frequency in peripheral blood cells (*closed box*).

Considering that plasma DNA samples can be easily contaminated with DNA originating from normal peripheral blood cells, we first analyzed the methylation status of the seven genes in peripheral blood cell samples from four noncancer patients. Methylation of *PPP1R3C* and *EFHD1* was rarely detected in peripheral blood cells, but the other five genes, *STOX2*, *EDIL3*, *COL4A2*, *TSPYL5*, and *ADAMTS1*, were frequently methylated in these cells (Fig.2). Given that false-positive results could potentially be obtained if these latter five genes were analyzed in plasma DNA samples, *PPP1R3C* and *EFHD1* were selected for subsequent analyses.

### Methylation of *PPP1R3C* and *EFHD1* in plasma and tumor samples from CRC patients

**Figure 3:**
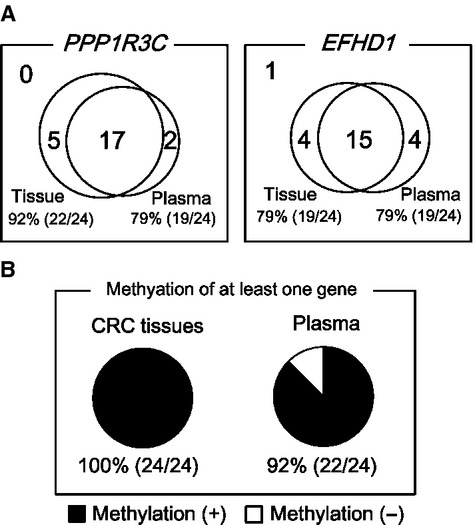
Screening of two genes using CRC tissue and corresponding plasma samples. (A) Methylation status in plasma and CRC tissue samples from 24 CRC patients. In another set of 24 CRC tissue samples than those in Figure1, *PPP1R3C* and *EFHD1* were confirmed to be frequently methylated at 92% (22/24) and 79% (19/24), respectively. The corresponding plasma samples were also frequently methylated at 79% (19/24) for each gene. (B) Frequency of the methylation of at least one gene. Among 24 patients, at least one of the two genes was methylated in 24 (100%) CRC tissue and 22 (92%) plasma samples.

*PPP1R3C* and *EFHD1* were analyzed using plasma samples from 24 CRC patients and their corresponding CRC tissue samples (Fig.3). *PPP1R3C* and *EFHD1* were methylated in 22 (92%) and 19 (79%) of the 24 CRC tissue samples, respectively. While these two genes were frequently methylated in 149 CRC tissue samples in the previous study, it was confirmed that they were also frequently methylated in this additional set of CRC tissue samples. When plasma DNA samples from these CRC patients were analyzed, *PPP1R3C* and *EFHD1* were frequently methylation-positive (+), at 79% (19/24) for each gene (Fig.3A).

When the two genes were combined, all 24 (100%) CRC tissue samples and 22 (94%) plasma DNA samples were methylation(+) for at least one of the two genes (Fig.3B). This suggested that high sensitivity could be obtained if these two genes were analyzed for CRC detection.

A small number of cases were methylation(+) in plasma DNA samples despite methylation(−) in CRC tissue samples. These might be due to unexpected methylation in peripheral blood cells contaminated in plasma samples or it might be due to heterogeneity of tumor tissues, that is, plasma DNA derived from a part of CRC might be methylated while the analyzed piece of CRC tissue might not be methylated.

### Comparison between CRC patients and noncancer patients

**Figure 4:**
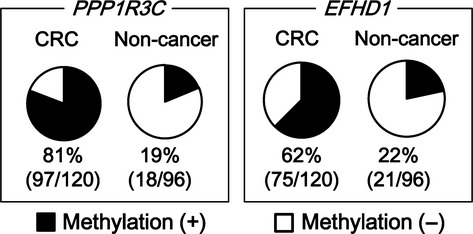
Methylation in plasma samples from 122 CRC patients and 96 noncancer patients. *PPP1R3C* was methylated in 81% (97/120) of CRC patients and in 19% (18/96) of noncancer patients (*P* = 6 × 10^−20^, Fisher's exact test). *EFHD1* was methylated in 62% (75/120) of CRC patients and in 22% (21/96) of noncancer patients (*P* = 3 × 10^−9^).

Next, *PPP1R3C* and *EFHD1* were analyzed using plasma samples from 120 CRC patients and 96 noncancer patients. *PPP1R3C* was methylated in 81% (97/120) of CRC patients (Fig.4), which was at a similar frequency determined for the initial 24 samples (Fig.3). The methylation(+) ratio for noncancer patients was 19% (18/96) (*P* = 6 × 10^−20^, Fisher's exact test). *EFHD1* was methylated in 62% (75/120) of CRC patients and in 22% (21/96) of noncancer patients (*P* = 3 × 10^−9^) (Fig.4).

**Figure 5:**
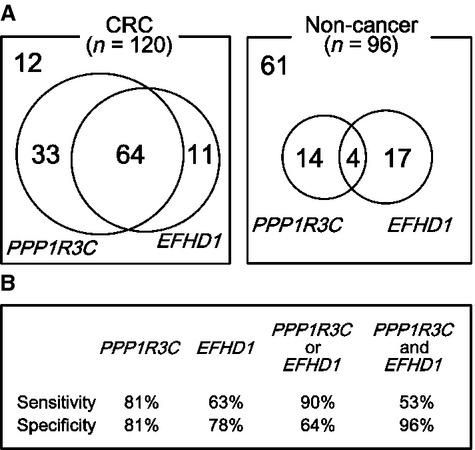
Sensitivity and specificity of the two methylation genes. (A) Combination of the two genes. Frequency of methylation of both genes was 53% (64/120) for CRC patients, but only 4% (4/96) for noncancer patients (*P* = 2 × 10^−16^, Fisher's exact test), giving high specificity. Frequency of methylation of at least one of the two genes was 90% (108/120) for CRC patients, but only 36% (35/96) for noncancer patients (*P* = 4 × 10^−17^), giving high sensitivity. (B) Sensitivity and specificity. Methylation of *PPP1R3C* gave better sensitivity and specificity, 81% and 81%, respectively, than did *EFHD1*. When the frequency of methylation of at least one gene was analyzed, sensitivity was increased to 90%. When the frequency of methylation of both genes was analyzed, specificity was as high as 96%.

If analyses of these two genes were combined, then at least one gene was methylated in 90% (108/120) of CRC patients and in 36% (35/96) of noncancer patients (*P* = 4 × 10^−17^). Both *PPP1R3C* and *EFHD1* genes were methylated in 53% (64/120) of CRC patients, but in only 4% (4/96) of noncancer patients (*P* = 2 × 10^−16^) (Fig.5A).

When a single gene was used for CRC detection using plasma samples, *PPP1R3C* gave better results than *EFHD1*. For *PPP1R3C*, 97 (81% sensitivity) of 120 CRC patients and 78 (81% specificity) of 96 noncancer patients were diagnosed correctly. The sensitivity and specificity could be improved when *EFHD1* was combined with *PPP1R3C*. If methylation of at least one gene was regarded as methylation(+), as many as 108 of 120 CRC patients would have been diagnosed correctly, with 90% sensitivity. If methylation of both genes was regarded as methylation(+), as many as 92 of 96 noncancer patients would have been diagnosed correctly, with 96% specificity, while the sensitivity would be 53% (Fig.5B).

### Comparison with protein markers, CEA and CA19-9

**Figure 6:**
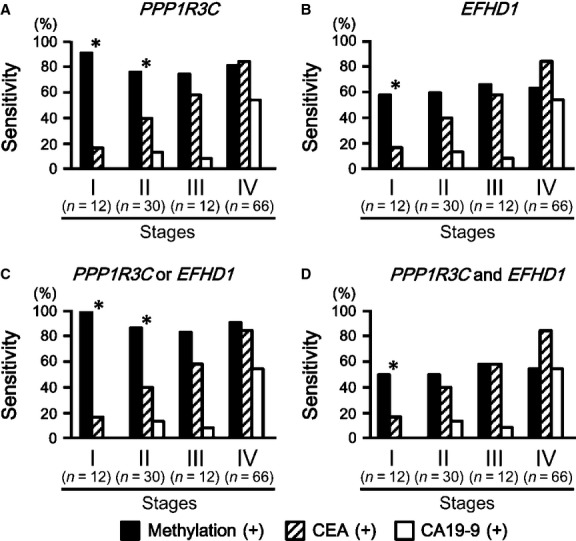
Comparison of the methylation genes with tumor markers, CEA and CA19-9. *Closed box*, methylation; *hatched box*, CEA; *open box*, CA19-9. While sensitivities for CEA and CA19-9 were 64% (77/120) and 34% (41/120), respectively, methylation showed a higher sensitivity, especially at early clinical stages. *P < 0.05, between methylation and CEA and between methylation and CA19-9. (A) Methylation of *PPP1R3C*. For stage I, 11 (92%) of 12 CRCs were *PPP1R3C* methylation(+), whereas 2 (17%) of 12 CRCs were CEA(+) (*P* = 3 × 10^−4^, Fisher's exact test), and 0% (0/12) were CA19-9(+) (*P* = 5 × 10^−6^). For stage II, 23 (77%) of 30 CRCs were *PPP1R3C* methylation(+), whereas 40% (12/30) were CEA(+) (*P* = 0.004) and 13% (4/30) were CA19-9(+) (*P* = 7 × 10^−7^). (B) Methylation of *EFHD1*. For stage I, 7 (58%) of 12 CRCs were *EFHD1* methylation(+) (*P* = 3 × 10^−4^ against CEA, *P* = 5 × 10^−6^ against CA19-9). (C) Methylation of at least one gene. For stage I, 12 (100%) CRCs were methylation(+) (*P* = 3 × 10^−5^ against CEA, *P* = 4 × 10^−7^ against CA19-9). For stage II, as many as 26 (87%) of 30 CRCs were methylation(+) (*P* = 2 × 10^−4^ against CEA, *P* = 6 × 10^−9^ against CA19-9). (D) Methylation of both *PPP1R3C* and *EFHD1*. For stage I, 6 (50%) CRCs were methylation(+) (*P* = 3 × 10^−5^ against CEA, *P* = 4 × 10^−7^ against CA19-9).

To evaluate the usefulness of the two methylation genes, their sensitivities were compared with two protein markers, CEA and CA19-9 (Fig.6). CEA and CA19-9 were positive in 64% (77/120) and 34% (41/120) of CRC cases, respectively. *PPP1R3C* methylation showed a higher sensitivity, 81% (97/120), than the two protein markers. At early clinical stages, sensitivity of *PPP1R3C* methylation was significantly higher than the protein markers (Fig.6A). For stage I CRC, 92% (11/12) samples were *PPP1R3C* methylation(+), whereas only 17% (2/12) were CEA(+) (*P* = 3 × 10^−4^, Fisher's exact test) and 0% (0/12) was CA19-9(+) (*P* = 5 × 10^−6^). For stage II CRC, 77% (23/30) were *PPP1R3C* methylation(+), whereas only 40% (12/30) were CEA(+) (*P* = 0.004) and 13% (4/30) were CA19-9(+) (*P* = 7 × 10^−7^).

Sensitivity of *EFHD1* methylation was also significantly higher than the protein markers for stage I CRC. Seven (58%) of 12 were *EFHD1* methylation(+), whereas 17% were CEA(+) (*P* = 3 × 10^−4^) and 0% was CA19-9(+) (*P* = 5 × 10^−6^) (Fig.6B).

When *EFHD1* methylation was combined with *PPP1R3C* analysis and methylation of at least one gene was regarded as methylation(+), the sensitivity at early clinical stages was further increased. All 12 (100%) were methylation(+) for stage I CRC (*P* = 3 × 10^−5^ against CEA and *P* = 4 × 10^−7^ against CA19-9). For stage II CRC, 87% (26/30) were methylation(+) (*P* = 2 × 10^−4^ against CEA and *P* = 6 × 10^−9^ against CA19-9) (Fig.6C). Even when methylation of both *PPP1R3C* and *EFHD1* was regarded as methylation(+), resulting in very high specificity, the sensitivity for stage I CRC was still significantly higher than that of the protein markers. Six (50%) of 12 CRCs were methylation(+), whereas 17% were CEA(+) (*P* = 3 × 10^−5^) and 0% was CA19-9(+) (*P* = 4 × 10^−7^).

### Comparison with other clinicopathological factors

Methylation status of *PPP1R3C* and *EFHD1* was compared with other clinicopathological factors including sex, age, tumor stage, and tumor locations (Tables 2 and 3). For both genes, methylation(+) and methylation(−) cases did not show significant difference in sex, age, tumor stage, presence or absence of neoadjuvant chemotherapy, and tumor locations.

## Discussion

Aberrant DNA methylation of promoter CpG islands is one of major epigenetic alterations in CRC 9,10. Some genes are commonly methylated in CRC regardless of epigenotypes and could possibly be utilized as CRC detection markers. Among these commonly methylated genes, ones methylated in normal colon samples or in peripheral blood cells were excluded. *PPP1R3C* and *EFHD1* were selected and subsequently analyzed using plasma DNA samples of 120 CRC and 96 noncancer patients, using methylation-specific PCR in combination with pyrosequencing for validation of specific amplification of methylated DNA. Detection of *PPP1R3C* methylation alone or its combination with *EFHD1* methylation in plasma DNA samples was found to show high sensitivity and specificity, and their sensitivities in early-stage CRCs were substantially higher than that of CEA and CA19-9.

In 2004, Müller et al. assessed *SFRP2* methylation in fecal DNA to diagnose CRC using MethyLight analysis; its sensitivity and specificity were as high as 77% and 77%, respectively, although they analyzed only 13 CRC and 13 control samples 5. In 2005, Chen et al. analyzed *VIM* methylation in fecal DNA from 94 CRC and 198 control samples using methylation-specific PCR; its specificity was as high as 90%, while sensitivity was 46% 24. As for methylation in plasma DNA, Lofton-Day et al. searched for CRC-specific methylated DNA in plasma and reported that the sensitivity and specificity of *TMEFF2*, *NGFR*, and *SEPT9* were 65% and 69%, 51% and 84%, and 69% and 86%, respectively 11. When *PPP1R3C* methylation was used alone in this study, its sensitivity (81%) and specificity (81%) were considerably high, compared with these reports.

Several groups analyzed *SEPT9* methylation in plasma samples for CRC detection. Some reports showed considerably high sensitivity (90–96%) and specificity (85–88%) 12,25, while other groups reported relatively lower sensitivity (48–72%) but higher specificity (86–95%) 26–28. In 2009, deVos et al. measured SEPT9 methylation using real-time PCR-based analysis, in which three independent experiments per sample were performed. High-sensitivity method, where at least one of three PCR was positive, resulted in 72% sensitivity and 86% specificity. But high-specificity method, where at least two of three PCRs were positive, resulted in 56% sensitivity and 95% specificity 27. This indicated that the results were dependent on the decision criteria, and that specificity would be increased by lowering sensitivity. Our results had similar tendencies. In high-sensitivity analysis where methylation of at least one gene was regarded as methylation(+), sensitivity improved to 90% while specificity was 64%. In high-specificity analysis where methylation of both the *PPP1R3C* and *EFHD1* genes was regarded as methylation(+), specificity improved to as high as 96% while sensitivity was 53%. These suggested that in addition to *SEPT9* methylation, *PPP1R3C* methylation alone or in combination with *EFHD1* methylation could be detection markers for CRC detection with high sensitivity and high specificity.

CRC is one of the leading causes of cancer deaths in the world, and diagnosis at an early onset followed by surgical intervention is currently the best way to cure the disease and decrease mortality. It is therefore important to develop detection markers to detect asymptomatic CRCs at earlier stages, while the sensitivities of CEA and CA19-9 were reported to be relatively low in early-stage CRCs 3. Our previous studies of DNA methylation in CRC and precancerous lesions revealed that accumulation of aberrant DNA methylation was mostly completed by the adenoma stage 15,29, suggesting the possible usefulness of assessing aberrant methylation in plasma DNA in detecting early-stage CRCs. Warren et al. reported that the sensitivity of *SEPT9* methylation was 71% for stage I CRCs 12. In another report, the sensitivity of *SEPT9* methylation was 60% for stage I CRCs, which could be increased to 84% using a high-sensitivity method 25. *PPP1R3C* methylation in this study gave a similar or even better results in detecting early-stage CRCs. The sensitivity of methylation of *PPP1R3C* alone was 92% for stage I CRCs. Using a more sensitive method to detect methylation of at least one of the *PPP1R3C* and *EFHD1* genes, the sensitivity increased to 100% for stage I CRCs. Even in a method with high specificity of 96%, the sensitivity of methylation of both genes was 50% for stage I CRCs, which was significantly higher than sensitivities of CEA (17%) and CA19-9 (0%). This indicated that detection of aberrant methylation in plasma DNA was a powerful method to diagnose CRC, especially for early-stage CRCs, and that *PPP1R3C* and *EFHD1* were useful biomarkers for the method.

A subgroup of methylation genes including CIMP markers were specifically hypermethylated in CIMP(+) high-methylation CRC, and methylation of these genes significantly associated with female, older age, and proximal tumor location 15. But the genes analyzed in this study were extracted from genes hypermethylated commonly in CRC regardless of epigenotypes, and methylation of these genes did not show significant correlation with sex, age, or tumor location 15. In good agreement with these previous observation, methylation of *PPP1R3C* and *EFHD1* in plasma DNA samples were detected commonly in CRC patients, regardless of sex, age, or tumor location (Tables 2 and 3).

**Table 3:** *EFHD1* methylation and clinicopathological factors

	Methylated	Unmethylated	*P-*value
Number	75	45	
Age (years)	67.0 ± 10.7	71.2 ± 12.3	0.5
Sex (male/female)	44/31	27/18	0.4
AJCC stage			1.0
I/II/III/IV	7/18/8/42	5/12/4/24	
NAC (yes/no)	13/62	7/38	0.7
Tumor location			0.4
Proximal (Ce/A/T)	26 (7/9/10)	15 (3/6/6)	
Distal (D/S)	20 (2/18)	17 (2/15)	
Rectum	29	13	

Age was shown by mean ± standard deviation. AJCC, American Joint Committee on Cancer; NAC, neoadjuvant chemotherapy. Tumor locations were classified into proximal colon including cecum (Ce), ascending (A) and transverse colon (T), distal colon including descending (D) and sigmoid colon (S), and rectum. *P*-values were analyzed using the Student's *t*-test for age and the Fisher's exact test for sex, stage, NAC, and tumor location.

In summary, detection of methylation of *PPP1R3C* alone or in combination with *EFHD1* in plasma DNA showed high sensitivity and specificity in CRC detection, and may be useful detection method for CRC, especially for early-stage CRCs.

## Supporting Information

**Figure S1.** Representative results of pyrosequencing analysis for methylation-specific PCR products of *PPP1R3C*.
