# Supplementary material for: Aberrant promoter methylation of PPP1R3C and EFHD1 in plasma of colorectal cancer patients
Source: Cancer Med. 2014 May 24;3(5):1235–45. doi: 10.1002/cam4.273 (PMC4302673; doi:10.1002/cam4.273)
Supplement: Supplementary file 1 — Figure S1. Representative results of pyrosequencing analysis for methylation-specific PCR products of PPP1R3C. [file cam40003-1235-SD1.pdf]

Supporting Fig. S1

Representative results of pyrosequencing analysis for methylation-specific PCR products of *PPP1R3C*. Bisulfite-treated genomic DNA was amplified using methylation-specific PCR, and specific amplification of the methylated allele was confirmed using pyrosequencing of the PCR product. (A) The methylation rate was expected to be as high as 60–100% when pyrosequencing was successful. If the methylation rate was low, which would have been due to unexpected amplification of the unmethylated allele in methylation-specific PCR, the sample would have been regarded as methylation(−). However, all the samples showed a methylation rate as high as 60–100% when the signal intensity was ≥5, and those samples were regarded as methylation(+). (B) When no signal was obtained in pyrosequencing, that should be due to no amplification in methylation-specific PCR, and the sample would be regarded as methylation(−). (C) When the signal intensity was too low to accurately calculate the methylation rate, this would be regarded as insufficient amplification by methylation-specific PCR. We set the threshold at 5; the sample was regarded as methylation(−) when the signal intensity was <5.

Fig. S1

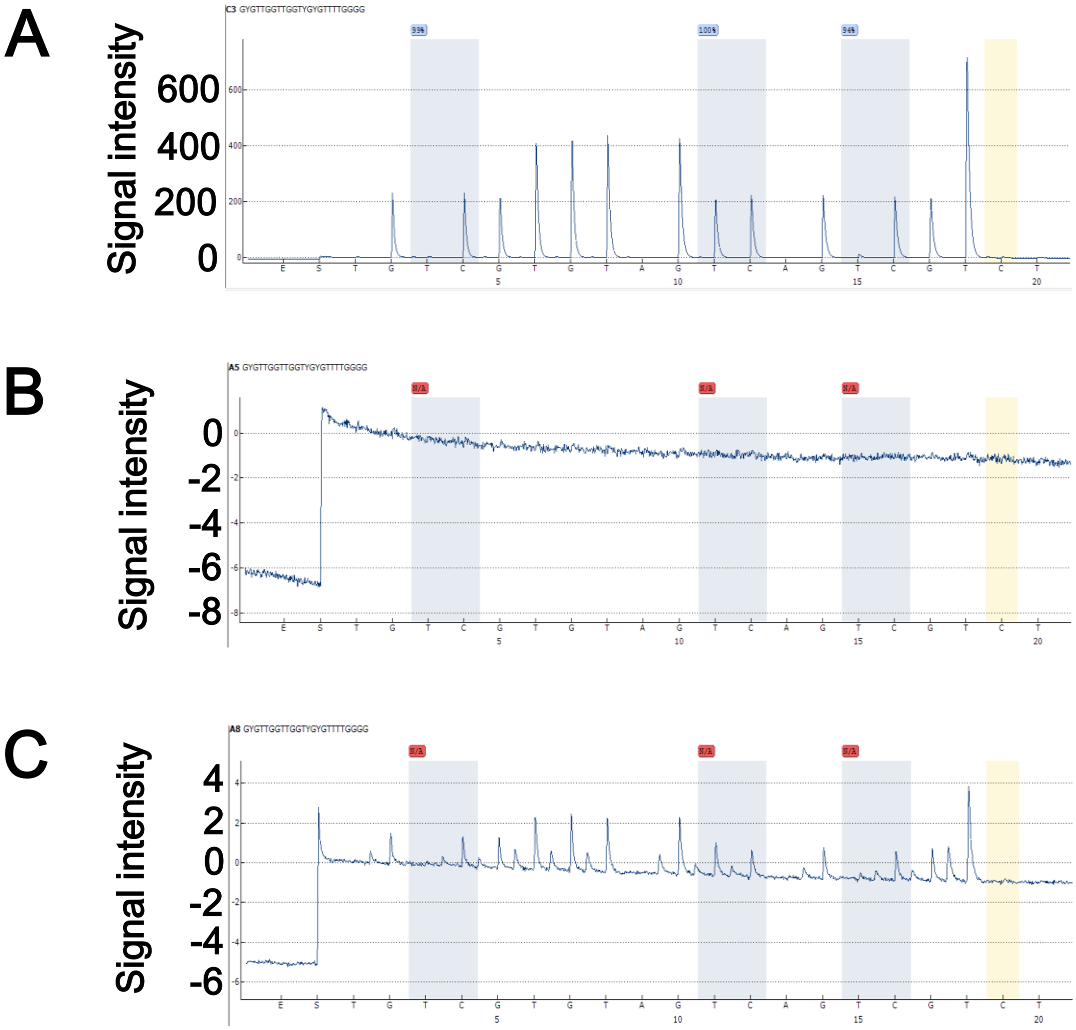

A
Signal intensity
600
400
200
0
C3 GYGTTGGTTGGTYGYGTTTTGGGG
99%
100%
94%
E S T G T C G T G T A G T C A G T C G T C T
5
10
15
20
B
Signal intensity
0
-2
-4
-6
-8
A5 GYGTTGGTTGGTYGYGTTTTGGGG
N/A
N/A
N/A
C
Signal intensity
4
2
0
-2
-4
-6
A8 GYGTTGGTTGGTYGYGTTTTGGGG
N/A
N/A
N/A
